# Effects of renal denervation on monocrotaline induced pulmonary remodeling

**DOI:** 10.18632/oncotarget.15154

**Published:** 2017-02-07

**Authors:** Qian Liu, Jiyang Song, Dasheng Lu, Jie Geng, Zhixin Jiang, Kai Wang, Bin Zhang, Qijun Shan

**Affiliations:** ^1^ Department of Cardiology, The First Affiliated Hospital of Nanjing Medical University, Nanjing, China; ^2^ Department of Cardiology, Gansu Provincial Hospital, Lanzhou, China

**Keywords:** renal denervation, monocrotaline, pulmonary remodeling

## Abstract

Pulmonary artery hypertension (PAH) is a rapidly progressive disorder, which leads to right heart failure and even death. Overactivity of the renin-angiotensin-aldosterone system (RAAS) and sympathetic nervous system accounts for the development and progression of PAH. The role of renal denervation (RDN) in different periods of PAH has not been fully elucidated. A single intraperitoneal injection of monocrotaline (MCT, 60 mg/kg) was used to induce pulmonary remodeling in male Sprague Dawley rats (*n* = 40). After 24-hour of MCT administration, a subset of rats underwent RDN (RDN_24h_, *n* = 10); after 2-week of MCT injection, another ten rats received RDN treatment (RDN_2w_, *n* = 10) and the left 20 rats were divided to MCT group with sham RDN operation (MCT, *n* = 20). Eight rats in Control group received intraperitoneal injection of normal saline (60 mg/kg) once and sham RDN surgery. After 35 days, tissue and blood samples were collected. Histological analysis demonstrated that the collagen volume fraction of right ventricle, lung tissue and pulmonary vessel reduced significantly in RDN_24h_ group but not in the RDN2w group, compared with MCT group. Moreover, the earlier RDN treatment significantly decreased SNS activity and blunted RAAS activation. Importantly, RDN treatment significantly improved the survival rate. In summary, earlier RDN treatment could attenuate cardio-pulmonary fibrosis and therefore might be a promising approach to prevent the development of PAH.

## INTRODUCTION

Pulmonary artery hypertension (PAH) is a complicated and fatal disease. Although progress achieved during last two decades with the introduction of oral medical therapies, the prognosis of PAH remains poor, especially in patients with advanced right heart failure [1]. It is imperative to seek potentially new therapies that prevent the development and progression of PAH. In the past few years, many experiments have indicated an important role of the renin-angiotensin-aldosterone system (RAAS) and sympathetic nervous system (SNS), in particular aldosterone [2–7]. Recently, de Man F.S. *et al*. found that the progression of PAH was accompanied by systemic RAAS overactivity [2].

We [8] and other investigators [9, 10] have reported that renal denervation (RDN) has the ability to reduce SNS activity and rebalance RAAS. It is reasonable to investigate the impact of RDN on PAH. And limited experiments have discussed the optimal time for RDN to treat PAH. Using a rat model of monocrotaline (MCT) induced pulmonary remodeling, we sought to study these isssues.

## RESULTS

### Right ventricular function

At day 35, echocardiography revealed significant increases in right ventricular anterior wall thickness (RVAW, 1.56 ± 0.20 mm vs. 1.00 ± 0.25 mm, *p* < 0.05), Non-filling time of right ventricle (NFT; r, 133.33 ± 30.97 ms vs. 106.44 ± 18.23 ms, *p* < 0.05), and pulmonary ejection time (PET, 88.89 ± 4.63 vs. 73.78 ± 5.59, p < 0.05) in MCT-induced PAH group compared with the RDN_24h_ group. Compared with MCT group, RVAW in RDN_2w_ group (1.03 ± 0.15 mm vs. 1.56 ± 0.20 mm, *p* < 0.05) was significantly reduced (Table 1).

**Table 1:** Echocardiographic parameters at day 35

Group	TV E/A	RVAW(mm)	NFT; r (ms)	PA VTI (mm)	PV peak Vel (mm/s)	PET (ms)
Control	0.61 ± 0.08	0.51 ± 0.05	97.22 ± 5.67	41.96 ± 11.01	–960.4 ± 98.0	73.78 ± 7.88
RDN_24h_	0.82 ± 0.27	1.00 ± 0.25^*#^	106.44 ± 18.23*	26.66 ± 2.97#	–816.6 ± 102.6^#^	73.78 ± 5.59*
RDN_2w_	0.89 ± 0.33	1.03 ± 0.15^*#^	124.58 ± 7.91^#^	26.10 ± 4.91#	–694.69 ± 94.5^#^	79.63 ± 8.98
MCT	1.17 ± 0.36^#^	1.56 ± 0.20^#^	133.33 ± 30.97^#^	23.75 ± 5.03#	–699.9 ± 108.1^#^	88.89 ± 4.63^#^

TV E: Tricuspid valve E wave velocity;

TV A: Tricuspid valve A wave velocity;

TV E/A: Ratio of tricuspid valve E' to A';

RVAW: Right ventricular anterior wall;

NFT; r: Non-filling time (right ventricle);

PA VTI: Pulmonary Artery velocity time integral;

PV peak vel: Pulmonary Artery peak velocity;

PET:Pulmonary Ejection Time.

*P < 0.05, vs. MCT; ^#^*P* < 0.05 vs. Control (*n* = 5 per group).

### Body weight (BW), heart weight (HW) and the ratio of HW/BW

Compared with Control, MCT significantly decreased the body weight (BW) (287.33 ± 57.08 g vs. 348.36 ± 46.25 g, *p* < 0.05) of the rats, while there was no significant differences between RDN (RDN_24h_ and RDN_2w_) groups and Control group. But RDN therapy had no influence on the ratio of HW/BW (Table 2).

**Table 2:** BW, HW, HW/BW

Group	Day	N	BW (g)	HW (mg)	HW/BW (mg/g)
Control	35	8	348.36 ± 46.25	1084.0 ± 159.55	3.12 ± 0.30
RDN_24h_	35	6	309.33 ± 22.29	1460.2 ± 133.42^#^	4.73 ± 0.38^#^
RDN_2w_	35	6	298.02 ± 49.97	1486.9 ± 91.70^#^	5.13 ± 1.04^#^
MCT	35	7	287.33 ± 57.08^#^	1405.2 ± 174.15^#^	5.06 ± 1.21^#^

N: the number of rats; BW: body weight; HW: heart weight; Num: number; HW/BW: the ratio of heart weight to body weight; MCT: monocrotaline.

*P < 0.05, vs. MCT; ^#^*P* < 0.05 vs. Control.

### Fibrosis of lung tissue and pulmonary vessel

**Figure 1:**
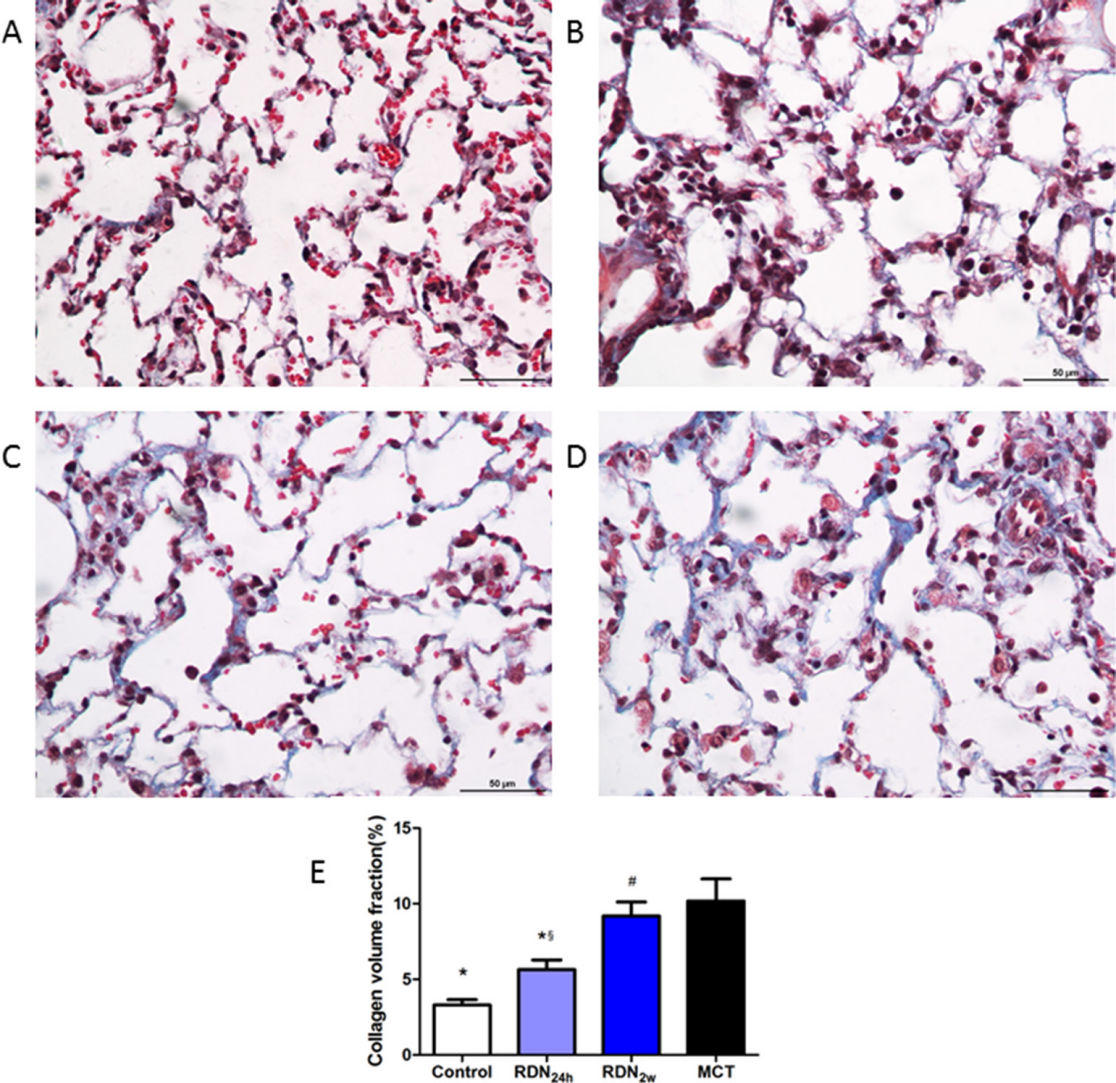
Effects of RDN on MCT-induced lung fibrosis histological section of lung Masson's Trichrome Staining (magnification× 400), the blue stands for fibrosis. (**A**) Control group (*n* = 8), (**B**) RDN_24h_ group (*n* = 6), (**C**) RDN_2w_ group (*n* = 6), (**D**) MCT group (*n* = 7). Comparison of collagen volume fraction (CVF,%) between four groups (**E**) *P < 0.05, vs. MCT; ^#^*P* < 0.05, vs. Control; ^§^*P* < 0.05, RDN_24h_ vs. RDN_2w_.

**Figure 2:**
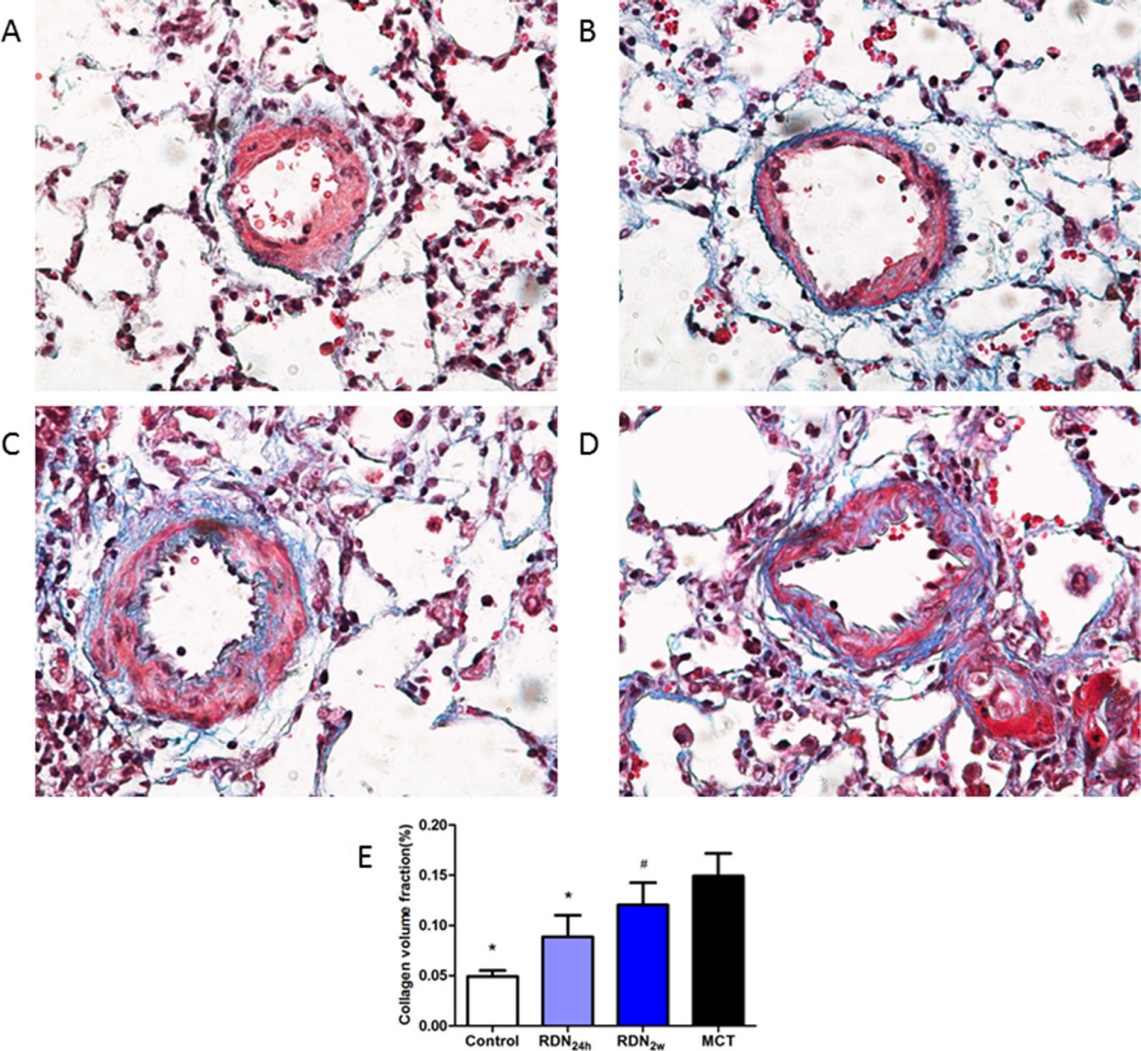
Effects of RDN on MCT-induced pulmonary vascular fibrosis histological section of pulmonary vascular Masson's Trichrome Staining (magnification× 400), the blue stands for fibrosis. (**A**) Control group (*n* = 8), (**B**) RDN_24h_ group (*n* = 6), (**C**) RDN_2w_ group (*n* = 6), (**D**) MCT group (*n* = 7). Comparison of collagen volume fraction (CVF,%) between four groups (**E**). *P < 0.05, vs. MCT; ^#^*P* < 0.05, vs. Control.

With regard to pulmonary fibrosis, collagen deposition was observed to be significantly higher in MCT challenged animals as measured by Masson's trichrome staining. These changes were completely manifested 35 days after MCT injection and without intervention with RDN procedure. Remarkably, RDN treatment after monocrotaline injection 24 hours reversed lung tissue fibrosis (5.64% ± 1.57% RDN_24h_ vs. 10.18% ± 3.90% MCT, *p* < 0.05) and pulmonary vascular fibrosis (8.87% ± 5.22% RDN_24h_ vs. 14.92% ± 5.52% MCT, *p* < 0.05). But, when we performed RDN procedure after monocrotaline injection for 2 weeks, the improvements in lung tissue fibrosis (9.19% ± 2.27% RDN_2w_ vs. 10.18% ± 3.90% MCT, *p* = 0.375) and pulmonary vascular fibrosis (12.05% ± 5.40% RDN_2w_ vs. 14.92% ± 5.52% MCT, *p* = 0.303) effects of RDN were blunted (Figures 1 and 2).

### Fibrosis of right ventricle and cross-sectional area of heart

**Figure 3:**
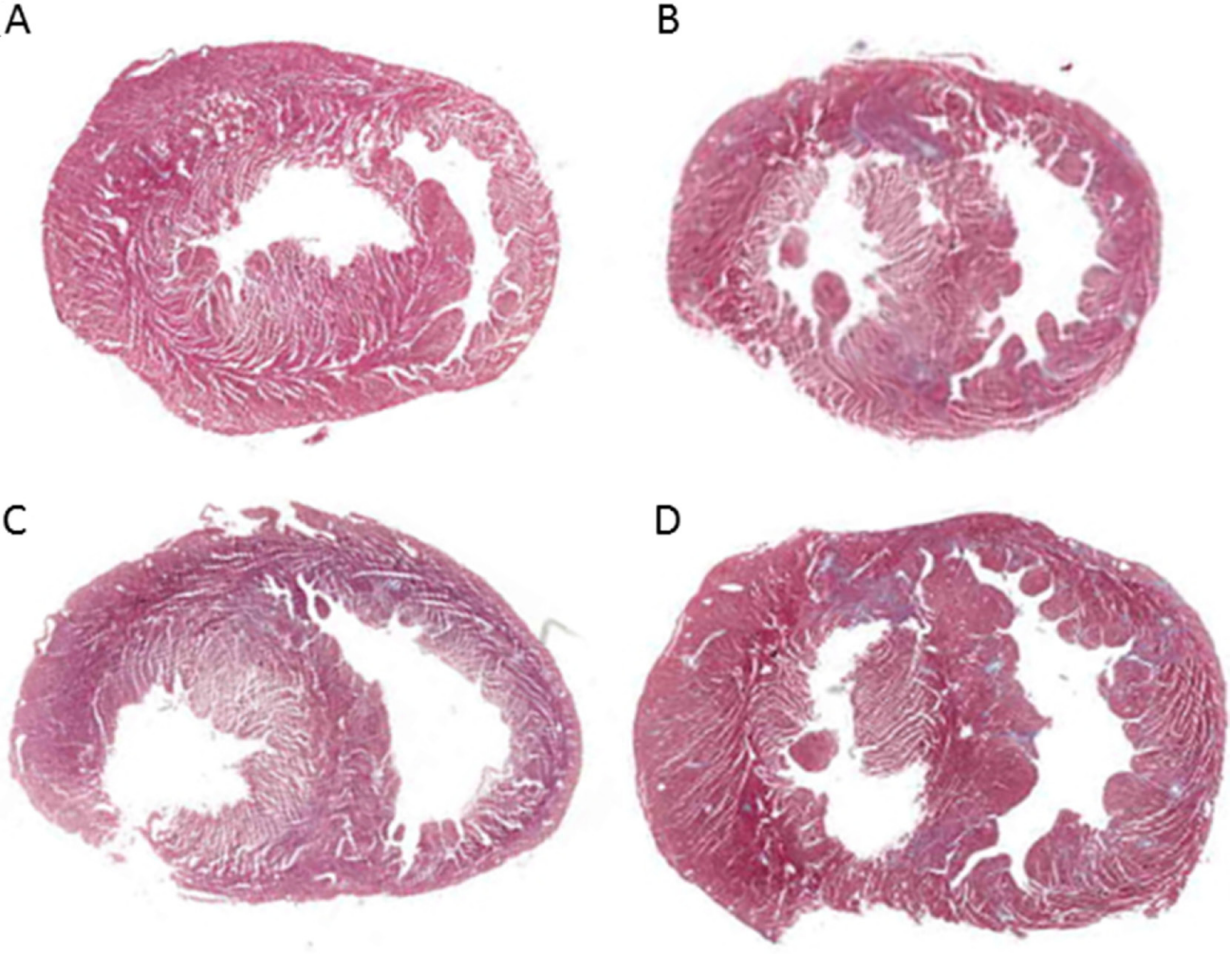
The Heart cross section (**A**) Control group (*n* = 8), (**B**) RDN24h group (*n* = 6), (**C**) RDN2w group (*n* = 6), (**D**) MCT group (*n* = 7). Chamber on the left side is left ventricular (LV), chamber on the right side is right ventricular (RV), tissue between two chambers is interventricular septal (IVS).

**Figure 4:**
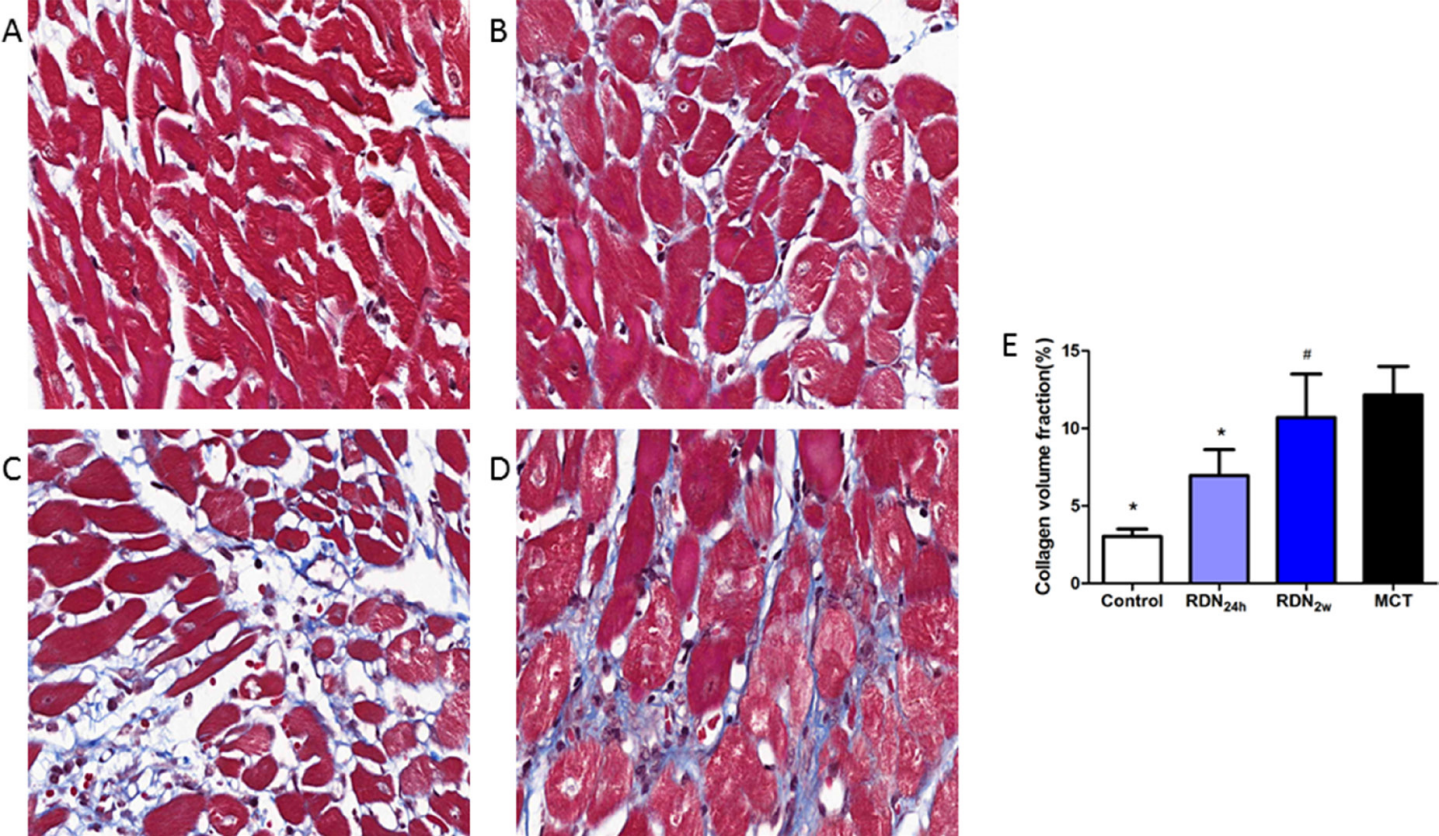
Right ventricular fibrosis histological section of right ventricular Masson's Trichrome Staining (magnification× 400), the blue stands for fibrosis. (**A**) Control group (*n* = 8), (**B**) RDN24h group (*n* = 6), (**C**) RDN2w group (*n* = 6), (**D**) MCT group (*n* = 7). Comparison of collagen volume fraction (CVF,%) between four groups (**E**). *P < 0.05, vs. MCT; ^#^*P* < 0.05, vs. Control.

RDN surgery performed in the earlier stage of PAH resulted in significantly reduced RVAW thickness (1.00 ± 0.25 mm vs. 1.56 ± 0.20 mm, *p* < 0.05) compared with MCT group (Figure 3). Monocrotaline injection results in pulmonary arterial hypertension and right heart failure, which were accompanied by myocardial and pulmonary fibrosis. The cross-sectional area of myocardial interstitial fibrosis was inhibited by earlier RDN therapy (11.39% ± 5.00% MCT vs. 6.96% ± 4.09% RDN_24h_, *p* < 0.05) which was increased by monocrotaline injection. However, there are no significant differences between RDN_2w_ group and MCT group (Figure 4).

### RDN reduces RAAS and SNS activity

**Figure 5:**
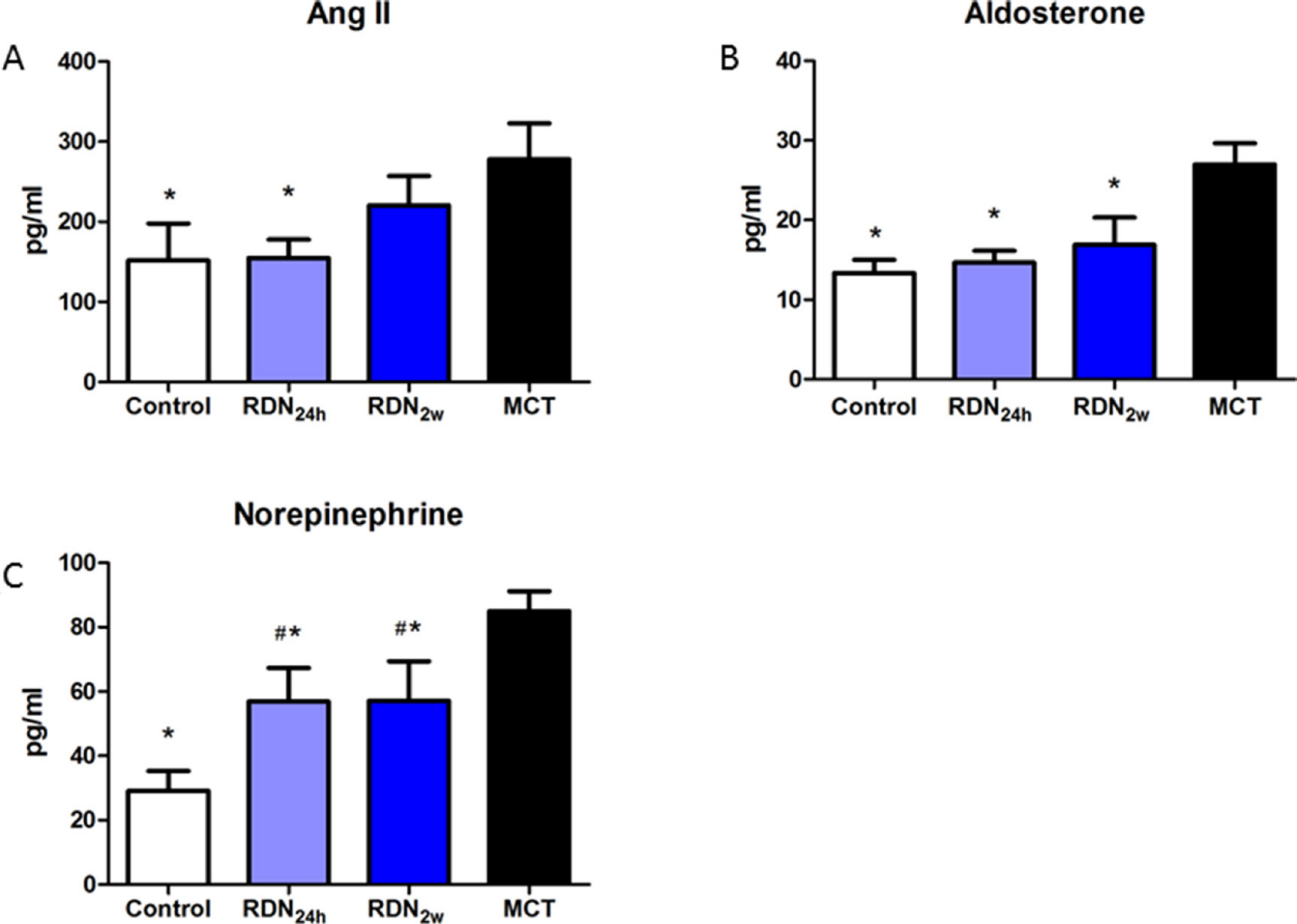
RDN Reduces RAAS and SNS activity effects of RDN on the changes of plasma Ang II (**A**) aldosterone (**B**) noradrenaline (**C**) levels in the four groups. *P < 0.05, vs. MCT group; ^#^*P* < 0.05, vs. Control group (*n* = 6 per group). Ang II, angiotensin II; RAAS, Renin–angiotensin–aldosterone system.

Ang II, which regulates fibroblast proliferation, differentiation and extracellular matrix deposition, is an important pro-fibrosis factor. Compared with MCT group, RDN_24h_ group significantly decreased plasma Ang II concentration (277.94 ± 110.00 pg/ml vs. 154.96 ± 55.98 pg/ml, *p* < 0.05), while only decreasing tendency of plasma Ang II concentration was observed in RDN_2w_ group. ALD, a member of RAAS, was also significantly reduced in RDN_24h_ (26.97 ± 6.53 pg/ml vs. 14.64 ± 3.70 pg/ml, *p* < 0.05) and RDN_2w_ (26.97 ± 6.53 pg/ml vs. 16.87 ± 8.44 pg/ml, *p* < 0.05) group. To response to overactive SNS, postganglionic neurons release high concentration NE, RDN blunted this increase. Compared with MCT, NE concentration significantly decreased in RDN_24h_ (84.98 ± 15.06 pg/ml vs. 56.90 ± 25.39 pg/ml, *p* < 0.05) and RDN_2w_ (84.98 ± 15.06 pg/ml vs. 57.16 ± 30.00 pg/ml, *p* < 0.05) group (Figure 5).

### Kaplan-Meier survival analysis

**Figure 6:**
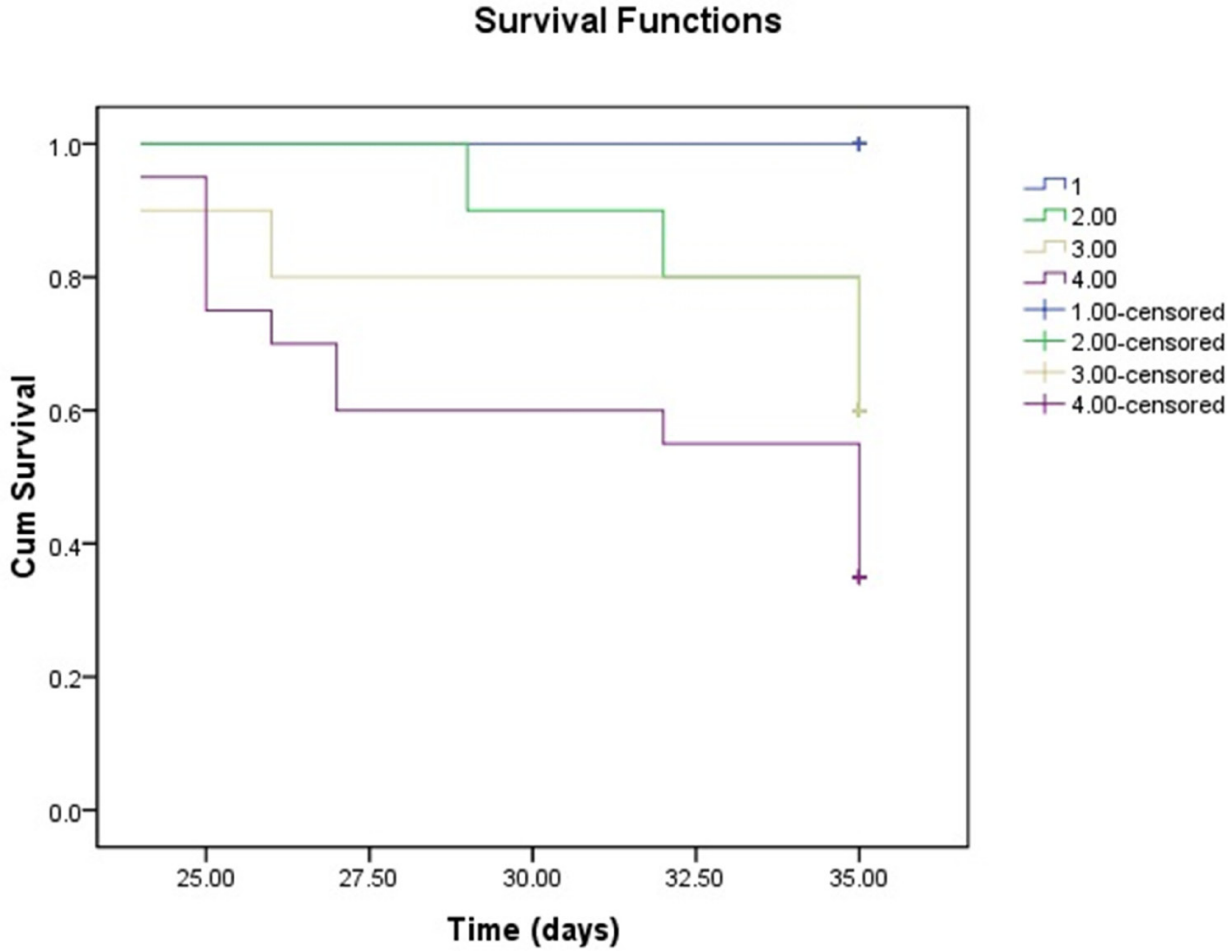
Kaplan-Meier survival analysis survival over the 35-day experiment was analyzed according to the daily recording of deaths by the standard Kaplan-Meier analysis with the log rank test

During the follow-up in the current experiment, 4 of 10 rats died both in the RDN_24h_ and RDN_2w_ group, and 13 of 20 animals died in the MCT group. None rats in the Control group died. On Kaplan–Meier survival analysis, after 35 days of follow-up, the rats treated with RDN (RDN_24h_ and RDN_2w_) had higher survival rate (60% vs. 35%) than MCT group (Figure 6).

## DISCUSSION

The present study mainly demonstrated that: 1) MCT induces remarkable fibrosis in lung tissue, pulmonary vascular and right ventricular; 2) RDN decreases the activity of RAAS and SNS; 3) RDN prevents lung tissue, pulmonary vascular and right ventricular fibrosis and results in a time-dependent improvement in cardio-pulmonary remodeling.

MCT-induced pulmonary remodeling is a process from lung tissue and pulmonary vascular fibrosis to pulmonary hypertension, and then causes right ventricular remodeling and failure [11, 12]. In this process, neurohormonal systems such as the RAAS and SNS are up-regulated [2]. At 35 days, echocardiography revealed significant increases in right ventricular anterior wall thickness (RVAW) in MCT-induced pulmonary remodeling group compared with control. Compared with Control, MCT significantly increased the the Ratio of HW/BW of the rats. We observed high concentration plasma NE, out-of-balanced RAAS axis, and excessive collagen deposition of cardio-pulmonary. While RDN surgery performed in the earlier stage resulted in significant attenuation of MCT-induced disease progression.

Pulmonary remodeling is a transitional phase to pulmonary hypertension and right heart failure. Thus, it is of great importance to find a way to slow or even reverse the progression of pulmonary remodeling. Velez-Roa S *et al*. have reported that the activity of SNS was increased in patients with pulmonary hypertension, and sympathetic activation participated in pulmonary arteriolar remodeling [7]. Previous data revealed that β-blockers could delay the progression toward right heart failure, and partially preserves right ventricular function in pulmonary hypertension [13, 14]. Linz D *et al*. had reported that RDN reduced tyrosine hydroxylase-positive sympathetic nerve staining and resulted in lower norepinephrine levels [15]. In recent studies, they had reported that pulmonary artery denervation induces permanent sympathetic nerve injury and subsequent improvements in hemodynamics and pulmonary remodeling in humans and animals with pulmonary hypertension [11, 16, 17]. In our prior study, we also found that RDN could regulate the expression of β1,2-receptors, which contribute to inhibit cardiac remodeling [8]. In accordance with these studies, we concluded that RDN significantly reduced NE level. The involvement of the RAAS in myocardial fibrosis is evident in several pathological conditions such as hypertensive heart disease [18], congestive heart failure [19] and myocardial infarction [20]. There is experimental and clinical evidence indicating that activated RAAS is involved in the pathophysiology of pulmonary hypertension [2, 21–23]. Ang II, the major effector peptide of the RAAS, promotes vasoconstriction, inflammation, proliferation and fibrosis within the lung [24, 25]. And plasma aldosterone levels were elevated in experimental models of pulmonary hypertension and humans with the disease [26, 27]. The persistently elevated level of aldosterone initiates signaling pathways that promote vascular remodeling, impair vascular reactivity, and contributes to right ventricular dysfunction [28]. We [8] and others [9, 10] had reported that RDN could reduce SNS activity and rebalance RAAS. In this regard, RDN has been of extreme significance when evaluating the effects of direct reducing SNS and RAAS activity. In our current study, we found a prominently beneficial effect of RDN in the earlier phase of disease process. What of particular interest was that RDN inhibited cardiopulmonary fibrosis and slowed down the progression of right heart failure. But treatment with RDN in the later period of MCT-induced pulmonary hypertension had no significant influence on pulmonary hemodynamics and remodeling of cardiopulmonary.

The prominent reversal of cardiopulmonary fibrosis is a remarkable and noteworthy effect of RDN treatment. Fibrosis is a common pathway to organ injury and failure [29]. We observed a 44.6% reduction of lung fibrosis, 40.5% reduction in pulmonary vascular fibrosis and 38.9% reduction in right ventricular fibrosis following RDN at the 35-day time point. As deposition of fibrous tissue in heart or lung is associated with the increasing risk of adverse cardiovascular events [30], the ability of RDN preventing fibrosis might contribute to a better prognosis of PAH.

Pulmonary hypertension is commonly associated with increased afterload, which causes maladaptive remodeling of the right ventricle, characterized by hypertrophy and fibrosis [31]. In addition, persistent pressure overload induces cardiomyocyte death and contractile dysfunction, which eventually leads to end-organ failure. The beneficial outcomes of RDN treatment were associated with decreased right ventricular remodeling and improved right heart function. But these beneficial effects were blunted when RDN performed in the later period.

Earlier RDN treatment in the process of PAH disease could improve right ventricular function and reduce cardio-pulmonary fibrosis, and it also improved survival rate in MCT-induced PAH. These experimental findings suggest that RDN should be further investigated as a potential approach for treating earlier stage of PAH.

### Study limitation

We should acknowledge the limitations in our study. Firstly, we failed to measure mean pulmonary arterial pressure at the end of this study. At day 35, we tried to test RV pressure and pulmonary artery pressure using right heart catheterization, but many rats in MCT group died when we opened their chests, and the collected data were too few to be analyzed. However, MCT-induced PAH is a well-established model, and both the mortality and echocardiographic data indicated that MCT had worked. Meanwhile, pulmonary remodeling was substantiated by histological analysis. Secondly, whether RDN is superior to oral drugs needs future investigation.

## MATERIALS AND METHODS

### Animals and experimental design

All procedures in this study were performed in accordance with the *Guide for The Care and Use of Laboratory Animals* (National Institutes of Health publication 8th edition, 2011) and were approved by the Nanjing Medical University Experimental Animal Care and Use Committee. The experiment was performed in male Sprague-Dawley rats weighing 200 ± 20 g (Nanjing Medical University Laboratory Animal Center), caged individually at controlled temperature and humidity with a 12-hour light/dark cycle. Echocardiography was performed at baseline and week 5. MCT injection was performed after echocardiography at baseline. Forty-eight male Sprague Dawley rats were randomized into 4 groups. Group 1 consisted of 8 rats that received normal saline and sham RDN procedure (Control). Group 2 comprised 10 rats that received MCT and RDN after 24 hour of intraperitoneally injected with 60 mg/kg MCT (MCT+RDN_24h_). Group 3 comprised 10 rats that received MCT and renal denervation after 2 weeks of intraperitoneally injected with 60 mg/kg MCT (MCT+RDN_2w_). Group 4 comprised 20 rats that received MCT and sham renal denervation procedure (MCT). At the end of the study, after the second echocardiography work-up and blood collection, all animals were euthanized with an overdose of pentobarbital sodium (200 mg/kg) by intraperitoneal injection.

### MCT-Induced PAH

Forty-eight male Sprague Dawley rats were randomly assigned to four groups: Control group (*n* = 8), MCT+RDN_24h_ group (*n* = 10), MCT+RDN_2w_ group (*n* = 10) and MCT group (*n* = 20). MCT-induced PAH rats were intraperitoneally injected with 60 mg/kg monocrotaline [32–34] (Sigma, Switzerland), which was dissolved in 1 mol/L HCL, the PH was adjusted to 7.4 and diluted with PBS.

### Renal denervation

With pentobarbital sodium (60 mg/kg intraperitoneal injection) anesthesia, bilateral renal denervation was performed in RDN_24h_ and RDN_2w_ group, whereas sham RDN procedure was performed in MCT and Control group. RDN was implemented as described previously [8]. Visible nerves along the renal arteries and veins were stripped and picked with 10× magnification. Chemical denervation was conducted by daubing the bilateral renal artery with 20% phenol solution in absolute alcohol. Then the arteries and veins were washed with isotonic saline. For sham RDN procedure, the operation was the same, but the renal arteries and veins were not isolated and the nerves were left intact.

### Echocardiography

Cardiac structure and function were evaluated by echocardiography with Vevo2100-a high resolution imaging system (VisualSonics, Canada) with a MS-250, 16.0–21.0 MHZ imaging transducer at baseline and week 5. All of the rats were anesthetized by aether during the process of echocardiography work-up.

### Histopathology

After perfusion with ice-cold PBS, the heart and lung were cut and fixed in 4% phosphate buffered formalin for 48–72 h at 4°C, then tissues were dehydrated and embedded in paraffin. The heart and lung tissues were fast frozen by liquid nitrogen, then moved to –80°C. Masson's trichrome staining was performed to detect right ventricular and lung fibrosis. Five fields of each sample were randomly selected and collagen volume fraction (CVF) was assessed by Image-Pro Plus 6.0.

### ELISA

Blood samples were obtained by EDTA-tubes, and then centrifuged at 3000 rpm at 4°C for 10 minutes to separate the plasma which stored at –80°C for later use. Plasma norepinephrine (NE), aldosterone (ALD) and angiotensin II (Ang II) levels at day 35 were measured using enzyme linked immunosorbent assay (ELISA) kits, according to the manufacturer's instructions (Uscn Life Science Inc, Wuhan, China).

### Kaplan-Meier survival analysis

Survival over the 35-day experiment was analyzed according to the daily recording of deaths by the standard Kaplan-Meier analysis with the log rank test.

### Statistics

Data are expressed as mean ± SEM and analyzed by SPSS 16.0 (SPSS Inc, Chicago, IL, USA). For two-group comparison, data were analyzed with two-tailed unpaired *t* tests. For multiple-groups comparisons, data were performed using one-way ANOVA with LSD test. *P* < 0.05 was considered statistically significant.

## CONCLUSIONS

In conclusions, RDN significantly prevents the overactivity of RAAS and SNS, and attenuates lung tissue, pulmonary vascular and right ventricular fibrosis in the earlier stage of pulmonary hypertension. Simultaneously, RDN improves the survival rate of PAH.
